# Spirulina Crude Protein Promotes the Migration and Proliferation in IEC-6 Cells by Activating EGFR/MAPK Signaling Pathway

**DOI:** 10.3390/md17040205

**Published:** 2019-04-01

**Authors:** Su-Jin Jeong, Jeong-Wook Choi, Min-Kyeong Lee, Youn-Hee Choi, Taek-Jeong Nam

**Affiliations:** 1 Institute of Fisheries Sciences, Pukyong National University, Busan 46041, Korea; Jsj941021@naver.com (S.-J.J.); wook8309@naver.com (J.-W.C.); 3633234@hanmail.net (M.-K.L.); 2 Department of Marine Bio-Materials & Aquaculture, Pukyong National University, Busan 48513, Korea; 3 Department of Food Science and Nutrition, Pukyong National University, Busan 48513, Korea

**Keywords:** cell cycle, EGFR signaling pathway, intestinal epithelial cells, MAPK signaling pathway, spirulina

## Abstract

Spirulina is a type of filamentous blue-green microalgae known to be rich in nutrients and to have pharmacological effects, but the effect of spirulina on the small intestine epithelium is not well understood. Therefore, this study aims to investigate the proliferative effects of spirulina crude protein (SPCP) on a rat intestinal epithelial cells IEC-6 to elucidate the mechanisms underlying its effect. First, the results of wound-healing and cell viability assays demonstrated that SPCP promoted migration and proliferation in a dose-dependent manner. Subsequently, when the mechanisms of migration and proliferation promotion by SPCP were confirmed, we found that the epidermal growth factor receptor (EGFR) and mitogen-activated protein (MAPK) signaling pathways were activated by phosphorylation. Cell cycle progression from G0/G1 to S phase was also promoted by SPCP through upregulation of the expression levels of cyclins and cyclin-dependent kinases (Cdks), which regulate cell cycle progression to the S phase. Meanwhile, the expression of cyclin-dependent kinase inhibitors (CKIs), such as p21 and p27, decreased with SPCP. In conclusion, our results indicate that activation of EGFR and its downstream signaling pathway by SPCP treatment regulates cell cycle progression. Therefore, these results contribute to the research on the molecular mechanism for SPCP promoting the migration and proliferation of rat intestinal epithelial cells.

## 1. Introduction

The surface of the intestinal epithelium is covered with a monolayer of intestinal epithelial cells (IECs), which digest food, absorb nutrients and prevent harmful external agents, such as toxins, allergens, and pathogens from entering the body [1,2]. It is important for the intestinal epithelium to maintain homeostasis, as it may be exposed to stronger physical stimuli compared to other body tissues [3]. When the surface of the intestinal epithelium is damaged, IECs adjacent to the injured region migrate to the damaged area and proliferate through cell division to maintain homeostasis of the intestinal mucosa [4]. However, intestinal disorders such as inflammatory bowel disease (IBD) cause repeated damage to the intestinal mucosal surface, resulting in IEC defects [5,6,7]. In this context, improving the migration and proliferation of IECs is a promising therapeutic strategy for intestinal disorders.

Previous studies have shown that growth factors such as transforming growth factor-α (TGF-α), hepatocyte growth factor (HGF), insulin-like growth factors I and II (IGF-I, IGF-II), epidermal growth factor (EGF), and glutamine [8,9,10,11,12,13,14] have protective and proliferative effects on IECs. In particular, EGF and its receptor (EGFR) play critical roles in the regulation of intestinal cell growth, survival, migration, proliferation, and differentiation [15]. In addition, EGF promotes maturation and maintenance of homeostasis in the early embryonic mouse gut [16]. The biological activity of EGF is mediated via binding to EGFR, leading to activation of receptor-associated tyrosine kinase, which phosphorylates tyrosine residues [17]. The mitogen-activated protein kinase (MAPK) signaling pathway is an EGFR-activated downstream signaling pathway that is activated in rapidly dividing IECs [18,19]. Moreover, previous studies have examined the role of the MAPK pathway in regulating the progression of cell cycle events, in particular controlling the G1-S phase transition by inducing expression of cyclins and Cdks required for S phase entry [20].

In previous studies, several marine algal proteins were shown to increase the phosphorylation of EGFR, promoting proliferation of IEC-6 cells [21,22,23]; therefore, we focused on the proliferative effect of marine algal protein on IECs. Marine algae comprise approximately half of global biodiversity and are rich sources of structurally diverse bioactive compounds with numerous biological activities [24,25]. Spirulina (*Arthrospira platensis*) is a marine/freshwater blue-green microalgae that is widely consumed as a nutritional supplement because it contains approximately 60–70% protein by dry weight, as well as other nutritional components such as minerals, vitamins, β-carotene and essential fatty acids, including γ-linolenic acid (GLA) [26,27].

Recently, increasing attention has been paid to the medicinal and pharmacological activities of spirulina, which include anti-cancer, anti-tumorigenic, anti-oxidant, and anti-obesity effects [28,29]. Furthermore, previous research has demonstrated that the population of beneficial *Lactobacillus* in the intestines of spirulina-fed rats increased 3-fold compared to controls without spirulina [30]. Although spirulina enhances intestinal health by promoting the growth of lactic acid bacteria in the intestine, the fundamental molecular mechanisms underlying its proliferative effect on IECs have not been fully elucidated. In previous research, EGFR demonstrated activity in regulating the migration and proliferation of IECs, and recent evidence indicates that spirulina crude protein (SPCP) increases the cellular viability of human dermal fibroblasts (CCD-986sk) by activating the EGFR/MAPK signaling pathway [31]. These results suggest that SPCP effectively regulates the EGFR/MAPK signaling pathway. Therefore, in this study, we examined the effects of SPCP on the EGFR and MAPK signaling pathways in rat IECs, i.e., IEC-6 cells.

## 2. Results

### 2.1. Electrophoresis Profiles of SPCP

**Figure 1:**
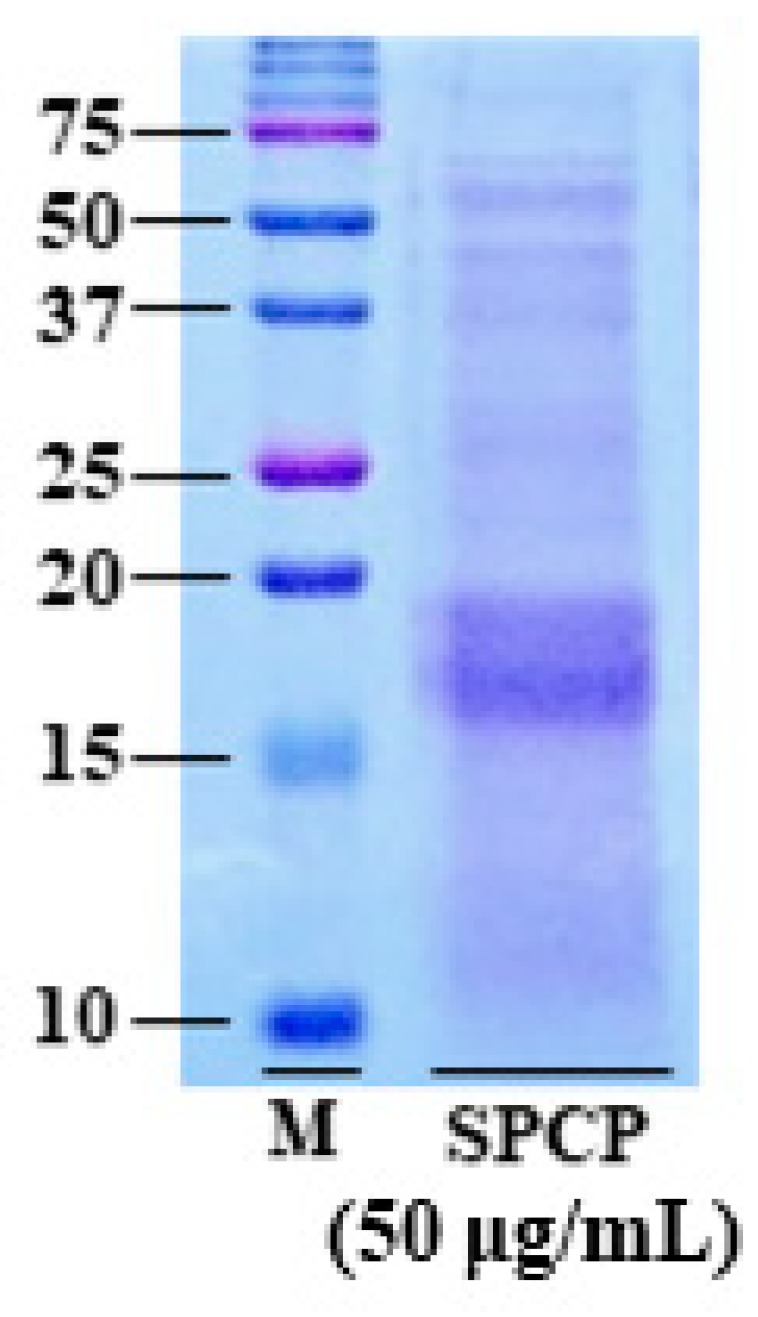
Electrophoresis profiles of SPCP. Crude protein extracted from spirulina (50 μg/mL) was applied to a 15% polyacrylamide gel and stained with Coomassie Brilliant Blue staining for protein. M, protein standard marker.

The amount of crude protein in the final SPCP preparation was measured by bicinchonicic acid (BCA) protein assay; it was 64.6 mg/mL in 100 mg. Moreover, to visualize protein bands of SPCP, 15% sodium dodecyl sulfate polyacrylamide gel electrophoresis (SDS-PAGE) and Coomassie Brilliant Blue staining were performed. As shown in Figure 1, it was confirmed that the presence of various protein bands on the 15% SDS-PAGE.

### 2.2. Effect of SPCP on Cell Migration and Proliferation in IEC-6 Cells

**Figure 2:**
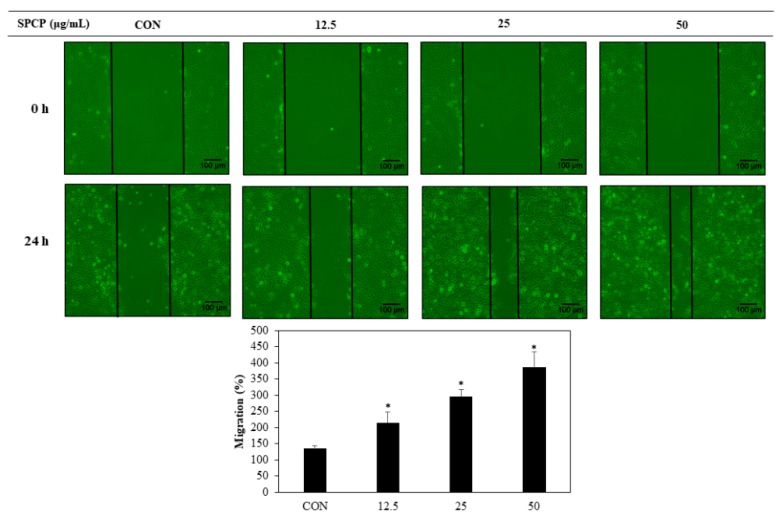
When IEC-6 cells were confluent in 6-well plates, uniform scratches were made using a sterilized tip. Then cells were serum-starved for 4 h and then treated with spirulina crude protein (SPCP) for 24 h. After washing with phosphate-buffered saline (PBS), the cells were photographed under a microscope at 100× magnification. Migration was assessed as the distance of movement between 0 h and 24 h, as measured using ImageJ software. The results presented are the means ± SD of three independent experiments. * *p* < 0.05 indicates a significant difference from the control group.

**Figure 3:**
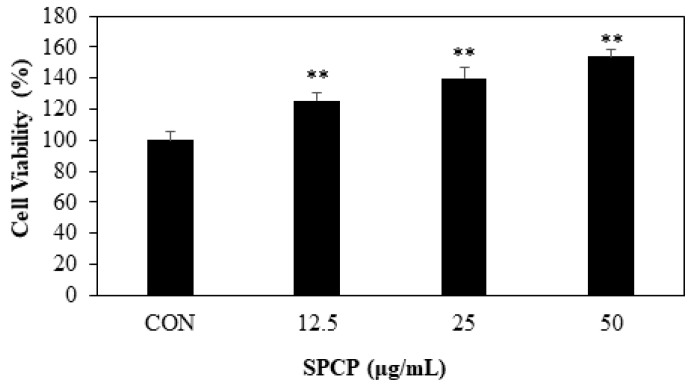
Effects of SPCP on the proliferation of IEC-6 cells. IEC-6 cells were seeded in 96-well plates at a density of 1 × 10^4^ cells/well. After the cells attached, they were serum-starved for 4 h and then treated with SPCP at the indicated concentrations for 24 h. The viability of cells was examined using the MTS assay. The results presented are the means ± SD of three independent experiments. ** *p* < 0.01 indicates a significant difference from the control group.

Cell migration induced by SPCP in IEC-6 cells was measured using a wound-healing assay. IEC-6 cells were seeded and cultured confluently in a 6-well plate, and then uniformly scratched. These IEC-6 cells were incubated for 24 h with SPCP at concentrations of 0, 12.5, 25, and 50 μg/mL. Compared to the control group without SPCP treatment, we observed that the SPCP treatment group showed significantly increased migration in a dose-dependent manner (Figure 2). To evaluate the SPCP-induced cell proliferation effect, an MTS assay was performed. IEC-6 cells were incubated for 24 h with SPCP. As shown in Figure 3, treatment with SPCP increased cell viability in a dose-dependent manner, and therefore the subsequent experiments were conducted in this concentration range.

### 2.3. Effect of SPCP Treatment on the EGFR and EGFR Adaptor Proteins

**Figure 4:**
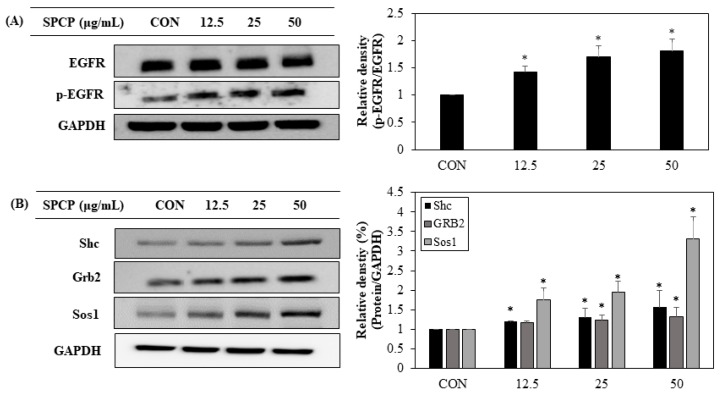
Effect of SPCP treatment on EGFR and EGFR adaptor protein expression in IEC-6 cells. (**A**) Protein expression levels of EGFR and p-EGFR were assessed through western blot analysis. (**B**) Protein expression levels of Shc, Grb2, and Sos1 were assessed through western blot analysis. GAPDH was used as an internal standard. The results are presented as means ± SD of three independent experiments. * *p* < 0.05 indicates a significant difference from the control group.

To investigate the mechanisms responsible for SPCP-induced proliferation of IEC-6 cells, the effects of SPCP on EGFR signaling-related proteins were examined through western blot analysis. As shown in Figure 4A, SPCP promoted protein expression levels of phosphorylation of EGFR significantly. In addition, treatment with SPCP upregulated protein expression levels of Shc, Grb2, and Sos1 in a dose-dependent manner compared to the control group untreated with SPCP (Figure 4B). These results indicate that SPCP treatment promotes EGFR signaling by stimulating EGFR phosphorylation and EGFR adaptor protein production.

### 2.4. Effect of SPCP Treatment on the ERK/MAPK Pathway

**Figure 5:**
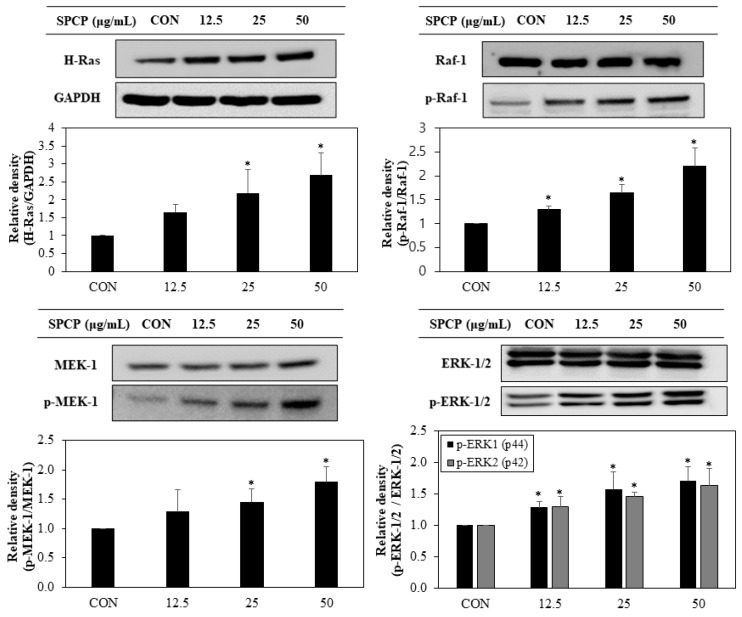
Effect of SPCP treatment on ERK/MAPK protein expression in IEC-6 cells. The protein expression levels of Ras, Raf-1, p-Raf-1, MEK-1, p-MEK-1, ERK1/2, and p-ERK1/2 in IEC-6 cells were assessed through western blot analysis. GAPDH was used as an internal standard. The results are presented as means ± SD of three independent experiments. * *p* < 0.05 indicates a significant difference from the control group.

To further clarify the downstream signals affected by EGFR activation, we investigated the expression of ERK/MAPK pathway. As shown in Figure 5, SPCP promoted upregulation of H-Ras and phosphorylation of Raf-1, MEK-1, and ERK-1/2 in a dose-dependent manner. These results indicate that SPCP treatment promotes ERK/MAPK signaling by activating the ERK/MAPK signaling pathway. Furthermore, we examined the effect of SPCP on the PI-3K/Akt. Our results (data not shown) suggest that SPCP does not affect PI-3K/Akt activation levels.

### 2.5. SPCP Promotes Cell Cycle Progression from G0/G1 to S Phages in IEC-6 Cells

**Figure 6:**
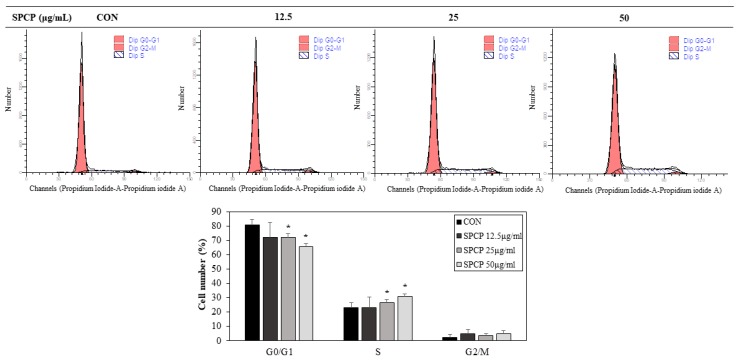
Effect of SPCP treatment on cell cycle progression in IEC-6 cells. IEC-6 cells were treated with SPCP at the indicated concentrations for 24 h, then fixed and stained with PI. Cell cycle analysis was performed using a BD FACSVerse flow cytometer, and the percentages of cells in the G0/G1, S, and G2/M phases were analyzed using the ModFit LT 2.0 program. The results are presented as means ± SD of three independent experiments. * *p* < 0.05 indicates a significant difference from the control group.

Cell proliferation is related to regulation of the cell cycle. To confirm whether the effect of SPCP on cell proliferation was accompanied by the progression of the cell cycle, IEC-6 cells were incubated with SPCP for 24 h and stained with propidium iodide (PI). As shown in Figure 6, SPCP treatment led to the decline of percentage of cells in G0/G1 phase from approximately 80.83% (±3.95) in the untreated control to 65.72% (±2.23) in the SPCP treated cells while the increase of the percentage of cells in the S-phase increased from approximately 23% (±3.48) to 30.98% (±1.81) in SPCP treated cells. In effect, SPCP caused a 15% decline, approximately, in the population of cells in the G0/G1 phase, with a 7% increase in the population of cells in the S-phase, which were statistically significant. These results indicate that SPCP-mediated cell proliferation occurred at the stage of G0/G1 to S progression.

### 2.6. Effect of SPCP Treatment on the Expression Cell Cycle Regulating Proteins

**Figure 7:**
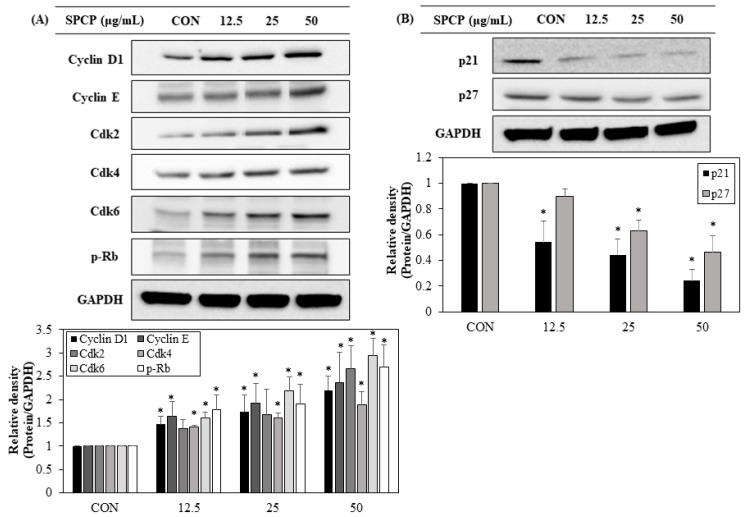
Effect of SPCP treatment on the expression of cell cycle regulatory proteins in IEC-6 cells. (**A**) Effect of SPCP treatment on cyclin and cyclin-dependent kinase (Cdk) protein expression in IEC-6 cells. The protein expression levels of cyclin D1, cyclin E, Cdk2, Cdk4, Cdk6, and p-Rb in IEC-6 cells were determined through western blot analysis. (**B**) Effect of SPCP treatment on cyclin-dependent kinase inhibitor (CKI) protein expression in IEC-6 cells. The protein expression levels of p21 and p27 in IEC-6 cells were assessed through western blot analysis. GAPDH was used as an internal standard. The results are presented as means ± SD of three independent experiments. * *p* < 0.05 indicates a significant difference from the control group.

Progression of the cell cycle is controlled by regulatory proteins that are activated during each phase of the cell cycle. To determine the proliferation mechanisms through which SPCP promotes cell cycle progression, the expression levels of cell cycle regulatory proteins were examined through western blot analysis. The levels of proteins that regulate the G0/G1 to S progression, including cyclin D1, cyclin E, Cdk 2, Cdk 4, Cdk 6 and p-Rb, were elevated (Figure 7A). In contrast the levels of proteins p21 and p27 that inhibit Cdk activity declined (Figure 7B). These results suggest that SPCP promotes proliferation of IEC-6 cells by modulating cell cycle regulatory proteins.

## 3. Discussion

Intestinal function depends on the homeostasis of the intestinal mucosal barrier, which is covered with a monolayer of IECs that regulate fundamental immunological functions [4]. Rapid recovery of the epithelial surface barrier following injuries due to normal digestion, toxic luminal substances, and inflammation are essential to maintaining this homeostasis [32]. To maintain the integrity of the barrier, IECs must migrate into a wound and proliferation to cover the damaged area [33]. One of the main aims of the present study was to investigate the influence of crude protein extracted from spirulina on the migration and proliferation of IEC-6 cells through cell cycle progression.

Previous studies have suggested that migration of epithelial cells initiates intestinal wound healing, as IECs move into the wound to cover the denuded mucosa over a few hours [2,34]. Our wound-healing assay results confirmed that SPCP increased the migration of IEC-6 cells. It has also been reported that the proliferation of IECs can restore the integrity of the intestinal mucosa within 24–48 h [35]. In the present study, the MTS assay showed that treatment with SPCP for 24 h effectively promoted IEC-6 cell proliferation in a dose-dependent manner. Thus, treatment with SPCP (12.5–50 μg/mL) for 24 h promotes intestinal epithelium restoration through migration and proliferation.

In multicellular organisms, cell proliferation is a complex process, with signaling pathways that include protein kinase cascades regulated primarily by growth factors [36]. To confirm the mechanism of migration and proliferation after SPCP treatment, we investigated protein expression of growth factors in IEC-6 cells. One regulatory mechanism that influences IEC migration and proliferation is EGFR signaling [15]. EGFR is widely expressed in mammalian epithelial tissues and activated by phosphorylation of the C-terminal domain, triggering downstream signaling pathways [37]. In previous studies, EGF application to murine IECs was reported to promote effective migration into a wounded area [38]. Furthermore, the phosphorylation level of EGFR increased, promoting proliferation of IEC-6 cells when exposed to proteins extracted from marine algae [21,22]. Similar to previous studies, we observed elevated expression of phosphorylated EGFR and its adapter proteins GRB2, Shc and Sos1 when IEC-6 cells were treated with SPCP. As EGFR mediates the regulation of IECs, these data indicate that SPCP treatment induced EGFR pathway activation.

Activated EGFR leads to the phosphorylation of downstream signaling pathways, such as the MAPK pathway [18]. Activation of Ras occurs primarily through EGFR adaptor complex proteins [39]. Previous research using caco-2/15 cells has shown that the MAPK pathway is an essential signaling pathway in the development of human intestinal cells [40]. In addition, glutamine, glucagon-like peptide 2, arginine vasopressin and apo-lactoferrin promote phosphorylation of ERK-1/2, leading to the proliferation of IECs [41,42,43,44]. Other studies have shown that ERK activation inhibited apoptosis, and conversely that ERK inactivation with MEK inhibitors increased apoptosis of IECs despite Ras activation [41]. Our study confirmed increased expression of Ras and phosphorylation of Raf-1, MEK-1, and ERK-1/2. These results support the previous finding that MAPK is crucial for the migration and proliferation of IECs and that MAPK-activated SPCP treatment is a key signaling pathway for IEC proliferation.

Prior studies have demonstrated that the MAPK signaling pathway plays a critical role in controlling cell growth and that ERK/MAPK activation during the G1-phase of the cell cycle is essential for entry to the S phase [45,46,47]. The cell cycle progresses through the G1, S, G2, and M phases, and this progression is mediated by interactions among cell cycle regulatory molecules including cyclins, cyclin-dependent kinases (Cdks), and Cdk inhibitory proteins [48]. The MAPK pathway is sequentially phosphorylated by Ras, promoting transcription of the cyclin D1 gene to initiate DNA replication [20]. Cyclin D increases due to activation of the EGFR and MAPK pathways and binds to Cdk4 and Cdk6 to form a complex, while cyclin E, which is expressed after cyclin D, forms a complex with Cdk2. Cyclin D and cyclin E phosphorylate retinoblastoma (Rb) protein and release transcription factor E2F, which activates the gene necessary for entry into the S phase [49,50,51]. Conversely, Cdk inhibitory proteins, such as p21 and p27, induce cell cycle arrest by preventing E2F from binding to Rb protein, accompanied by inhibition of IEC-6 cell growth [52,53]. On the other hand, Cdk inhibitory proteins have been reported to induce intestinal epithelial differentiation [54,55]. In the present study, SPCP treatment increased the proportion of S-phase cells in flow cytometric analysis, and the data indicated that SPCP promoted the G1/S transition through the EGFR and MAPK pathways. In addition, we confirmed up-regulation of cyclin D1, cyclin E, Cdk 2, Cdk 4, Cdk 6, and p-Rb and down-regulation of cyclin-dependent kinase inhibitors (CKIs), including p21 and p27. Taken together, our results show that SPCP activity promoted the G1/S transition in the cell cycle by regulating cyclins, Cdks, and CKIs.

In conclusion, this study revealed that SPCP plays a role in promoting the migration and proliferation of IEC-6 cells and activating the EGFR and MAPK signaling pathways. In addition, the results provide molecular evidence that the proliferative effects of SPCP are regulated in part by cell cycle progression. Overall, SPCP may be a promising pharmacological agent for maintaining intestinal epithelial homeostasis.

## 4. Materials and Methods

### 4.1. Preparation of SPCP

SPCP was prepared by using the method of Liu et al. [31]. To extract SPCP, spirulina powder was purchased from New Zealand Nutritionals Ltd. (Burnside, Christchurch, New Zealand). Spirulina powder (40 g) was diluted with 1 L distilled water and stirred for 4 h at room temperature. The resultant spirulina solution was centrifuged at 2400× *g* at 4 °C for 10 min. Then three volumes of ethanol were added to the supernatant and kept in 4 °C for 24 h. After 24 h, the solution was centrifuged again and then filtered and concentrated using rotary evaporation at 40 °C. The supernatant was added to 80% ammonium sulfate and stirred for 24 h at 4 °C for protein precipitation. The precipitate was added to distilled water, and then dialyzed using a 3500-Da MW Spectra/Por membrane (Spectrum Labs, Rancho Dominguez, CA, USA) for 72 h at 4 °C. The dialysate was concentrated using rotary evaporation and then freeze-dried to produce a powder. The powder was stored at −70 °C until use, and referred to as SPCP. The SPCP were solubilized with distilled water and quantified by bicinchoninic acid (BCA) protein assay kit (Pierce; Thermo Fisher Scientific, Rockford, IL, USA) for use in the subsequent experiments.

### 4.2. SDS-PAGE and Coomassie Brilliant Blue Staining

To visualize proteins of SPCP, SDS-PAGE and Coomassie Brilliant Blue staining were performed. Firstly, the SPCP were mixed in 5X sample loading buffer (50 mM Tris-HCl, 2% SDS, 10% Glycerol, 0.02% Bromophenol blue and 5% 2-mercaptoethanol), boiled for 5 min and separated through 15% SDS-PAGE [56]. Dual color marker (BIO-RAD, Richmond, CA, USA) was used for the molecular mass of the protein’s determination. After SDS-PAGE, the gels were stained with Coomassie Brilliant Blue for 1 h and then washed with the de-staining solution until the protein bands visualized.

### 4.3. Cell Culture

IEC-6 rat small IECs (ATCC CRL-1592; American Type Culture Collection, Manassas, VA, USA) were cultured in Dulbecco’s modified Eagle’s medium (DMEM) (Gibco; Thermo Fisher Scientific, Waltham, MA, USA) supplemented with 10% fetal bovine serum (FBS; Gibco; Thermo Fisher Scientific, Waltham, MA, USA), 100 units/mL penicillin, and 100 μg/mL streptomycin (Gibco; Thermo Fisher Scientific, Waltham, MA, USA) at 37 °C in an atmosphere of 5% CO_2_. IEC-6 cells were grown to 70–80% confluence in 100-mm culture dishes and trypsinized for subculturing every 3 to 4 days.

### 4.4. Wound-Healing Assay

Cell migration was analyzed using wound-healing assays, as described previously [57]. IEC-6 cells were cultured in 6-well plates with DMEM supplemented with 10% FBS. When the cells were confluent, uniform scratches were made using sterilized 200-μL pipette tips. After washing three times with phosphate-buffered saline (PBS), the cells were photographed under a phase contrast microscope at 100× magnification (0 h). The cells were maintained in serum-free medium (SFM) for 4 h, and then treated with SPCP for 24 h. After washing three more times with PBS, the cells were photographed under the same conditions (24 h). The distances were measured using ImageJ software (version 4.16; National Institutes of Health, Bethesda, MD, USA). 

### 4.5. MTS Assay

To evaluate cellular viability, the CellTiter 96 Aqueous Non-Radioactive Cell Proliferation Assay (Promega, Madison, WI, USA) was used. Cells were seeded at a density of 1 × 10^4^ cells/well in 96-well plates and cultured using DMEM supplemented with 10% FBS. After 24 h of incubation, the cells were maintained in SFM for 4 h, and then the medium was replaced with SFM containing SPCP for 24 h at 37 °C. MTS solution was added and the cells were incubated at 37 °C for 30 min. The absorbance at 490 nm was measured using a Gen5 ELISA microplate reader (Bio-Tex, Houston, TX, USA). The OD490 values of the control cells were designated as 100%.

### 4.6. Preparation of Cell Lysate

To prepare total cell lysates, IEC-6 cells were cultured to 40–50% confluence and then maintained in SFM. After 4 h, SFM containing SPCP (0, 12.5, 25, and 50 μg/mL) was added to the cells, which were incubated for 24 h. Cells were washed twice with PBS and lysed with radioimmunoprecipitation assay (RIPA) buffer containing Protease Arrest protease inhibitors (G-Biosciences, St. Louis, MO, USA) on ice. Cell lysates were collected on ice, centrifuged at 18,000× *g* for 10 min at 4 °C, and then the supernatant was analyzed using bicinchoninic acid (BCA) reagent for western blotting.

### 4.7. Western Blotting Analysis

Equal amounts of protein sample (30 µg) were separated through 7–12.5% SDS-PAGE and transferred to polyvinylidene fluoride (PVDF) membranes. The membranes were activated with methanol and blocked with 1% bovine serum albumin (BSA) in TBS-T (10 mM Tris-HCl, 150 mM NaCl and 0.1% Tween-20) at room temperature, then incubated with primary antibodies (Table 1) at 4 °C. After overnight incubation, the membranes were washed with TBS-T and incubated with secondary antibodies for 2 h at room temperature. The secondary antibodies were used anti-mouse Ig G (cat. no. 7076S; Cell Signaling Technology, Inc., Beverly, MA, USA), anti-rabbit Ig G (cat. no. 7074S; Cell Signaling Technology, Inc., Beverly, MA, USA) and donkey anti-goat Ig G (cat. no. A50-101p; Bethyl Laboratories, Inc., Montgomery, TX, USA) diluted 1: 10,000 in TBS-T. The enhanced chemiluminescence (ECL) kit (Thermo Fisher Scientific, Rockford, IL, USA) was used to detect immunoreactivity. Densitometric analysis was conducted through triplicate experiments and analyzed using Multi-Gauge software (version 3.0; Fujifilm Life Science, Tokyo, Japan).

### 4.8. Cell Cycle Analysis

Cell cycle analysis was performed using the BD Cycletest Plus DNA kit (BD Biosciences, San Jose, CA, USA). IEC-6 cells were cultured in 100-mm culture dishes to 40–50% confluence and treated with SPCP (0, 12.5, 25, and 50 μg/mL) for 24 h. After the treatment period, cells were trypsinized and the cell number was adjusted to 1.0 × 10^6^ cells/mL using a hemocytometer. Cell pellets were suspended in 250 μL trypsin buffer at room temperature for 10 min and then 200 μL trypsin inhibitor and RNase buffer were added for 10 min. Finally, 200 μL of ice cold PI stain was added and the cells were placed in the dark for 10 min. Stained cells were analyzed using a flow cytometer (BD FACSVerse; BD Biosciences, San Jose, CA, USA) and data from at least 10,000 single-cell events were acquired for each group. The percentages of cells in the G1, S, and G2 phases were determined using ModFit software (version 2.0; Verity Software House Inc., Topsham, ME, USA).

### 4.9. Statistical Analysis 

Mean values were analyzed using the SPSS program (version 10.0; SPSS Inc., Chicago, IL, USA). Values are presented as means ± standard deviations. The data were compared statistically using one-way analysis of variance (ANOVA) followed by Duncan’s multiple range test.

## Figures and Tables

**Table 1:** Primary antibodies used in the present study.

Primary Antibody	Manufacturer	Species	Dilution
Cdk2	Santa Cruz Biotechnology, sc-163	rabbit	1:1000
Cdk4	Santa Cruz Biotechnology, sc-166373	mouse	1:1000
Cdk6	Santa Cruz Biotechnology, sc-177	rabbit	1:1000
Cyclin D1	Santa Cruz Biotechnology, sc-8396	mouse	1:1000
Cyclin E	Santa Cruz Biotechnology, sc-481	rabbit	1:1000
EGFR	Santa Cruz Biotechnology, sc-373746	mouse	1:500
ERK-1	Santa Cruz Biotechnology, sc-94	mouse	1:1000
GAPDH	Santa Cruz Biotechnology, sc-137179	mouse	1:1000
Grb2	Santa Cruz Biotechnology, sc-8034	mouse	1:1000
H-Ras	Santa Cruz Biotechnology, sc-520	rabbit	1:1000
MEK-1	Santa Cruz Biotechnology, sc-219	mouse	1:1000
p21	Santa Cruz Biotechnology, sc-271532	mouse	1:1000
p27	Santa Cruz Biotechnology, sc-56338	mouse	1:1000
p-EGFR	Santa Cruz Biotechnology, sc-12351	goat	1:500
p-ERK-1/2	Santa Cruz Biotechnology, sc-7383	mouse	1:1000
p-MEK-1	Santa Cruz Biotechnology, sc-81053	mouse	1:1000
p-Raf-1	Santa Cruz Biotechnology, sc-271929	mouse	1:1000
p-Rb	Santa Cruz Biotechnology, sc-377528	mouse	1:1000
Raf-1	Santa Cruz Biotechnology, sc-227	rabbit	1:1000
Shc	Santa Cruz Biotechnology, sc-398289	mouse	1:1000
Sos1	Santa Cruz Biotechnology, sc-259	rabbit	1:1000
